# Mitogenomes from Egyptian Cattle Breeds: New Clues on the Origin of Haplogroup Q and the Early Spread of *Bos taurus* from the Near East

**DOI:** 10.1371/journal.pone.0141170

**Published:** 2015-10-29

**Authors:** Anna Olivieri, Francesca Gandini, Alessandro Achilli, Alessandro Fichera, Ermanno Rizzi, Silvia Bonfiglio, Vincenza Battaglia, Stefania Brandini, Anna De Gaetano, Ahmed El-Beltagi, Hovirag Lancioni, Saif Agha, Ornella Semino, Luca Ferretti, Antonio Torroni

**Affiliations:** 1 Dipartimento di Biologia e Biotecnologie "L. Spallanzani", Università di Pavia, Pavia, Italy; 2 School of Applied Sciences, University of Huddersfield, Queensgate, Huddersfield, United Kingdom; 3 Dipartimento di Chimica, Biologia e Biotecnologie, Università di Perugia, Perugia, Italy; 4 Istituto di Tecnologie Biomediche, Consiglio Nazionale delle Ricerche, Segrate (Milano), Italy; 5 Fondazione Telethon, Milano, Italy; 6 Animal Production Research Institute (APRI), Ministry of Agriculture, Cairo, Egypt; 7 Department of Animal Production, Faculty of Agriculture, Ain Shams University, Cairo, Egypt; University of Florence, ITALY

## Abstract

**Background:**

Genetic studies support the scenario that *Bos taurus* domestication occurred in the Near East during the Neolithic transition about 10 thousand years (ky) ago, with the likely exception of a minor secondary event in Italy. However, despite the proven effectiveness of whole mitochondrial genome data in providing valuable information concerning the origin of taurine cattle, until now no population surveys have been carried out at the level of mitogenomes in local breeds from the Near East or surrounding areas. Egypt is in close geographic and cultural proximity to the Near East, in particular the Nile Delta region, and was one of the first neighboring areas to adopt the Neolithic package. Thus, a survey of mitogenome variation of autochthonous taurine breeds from the Nile Delta region might provide new insights on the early spread of cattle rearing outside the Near East.

**Methodology:**

Using Illumina high-throughput sequencing we characterized the mitogenomes from two cattle breeds, Menofi (N = 17) and Domiaty (N = 14), from the Nile Delta region. Phylogenetic and Bayesian analyses were subsequently performed.

**Conclusions:**

Phylogenetic analyses of the 31 mitogenomes confirmed the prevalence of haplogroup T1, similar to most African cattle breeds, but showed also high frequencies for haplogroups T2, T3 and Q1, and an extremely high haplotype diversity, while Bayesian skyline plots pointed to a main episode of population growth ~12.5 ky ago. Comparisons of Nile Delta mitogenomes with those from other geographic areas revealed that (i) most Egyptian mtDNAs are probably direct local derivatives from the founder domestic herds which first arrived from the Near East and the extent of gene flow from and towards the Nile Delta region was limited after the initial founding event(s); (ii) haplogroup Q1 was among these founders, thus proving that it underwent domestication in the Near East together with the founders of the T clades.

## Introduction

The domestication of the wild aurochs (*Bos primigenius*) was a major element of the Neolithic transition. Archeozoological evidence indicates that taurine cattle were initially domesticated somewhere in the upper Euphrates Valley between 11 and 10 thousand years (ky) ago, whereas zebuine cattle (*Bos indicus*) arose from an independent domestication event that occurred about two millennia later in the Indus Valley [1–4]. As for genetic evidence, despite some relevant data from recent genome-wide studies [5, 6], most of the available fine-scale details derive from phylogenetic studies of the maternally inherited mitochondrial DNA (mtDNA). Since the beginning these studies involved modern samples [7–9] as well as ancient specimens [10–15] generally focusing on a control-region segment (often only 240 bp) which has been sometimes expanded in more recent studies [16–18]. This approach revealed that modern taurine mtDNAs cluster within a number of closely related branches, initially termed T, T1, T2, T3 and T4, and that these branches were phylogenetically distant from the P haplotypes observed in European aurochs specimens [7, 10, 14] and even more distant from the haplogroup I mtDNAs (I1 and I2) that characterize modern zebuine cattle [7, 19–21]. Most importantly, frequency and geographic distributions of the T lineages were very compatible with the scenario of a single ancestral population source in the Near East and a later spread of *Bos taurus* following its domestication event in that area [7], even though alternative models were also proposed to explain some peculiar features in the geographic distributions of T1 [10], T3 [11] and T4 [8].

In more recent years, mainly due to technological advancements, a slow shift towards analyses carried out at the whole mitochondrial genome (mitogenome) level has occurred [22–29]. The whole mitogenome sequencing approach has revealed the fine phylogenetic structure of what is now termed "macro-haplogroup T". This is made up of two clades, T1'2'3 and T5 [22, 23]. The latter was a previously unknown haplogroup, while T1’2’3 is formed by the previously defined T1, T2 and T3. Haplogroup T4 turned out to be a derived sub-clade within T3. Furthermore, the use of mitogenome sequences allowed for more precise estimates of haplogroup coalescence times. The age of super-haplogroup T was estimated at ∼16 ky, and those of haplogroups T1, T2, T3 and T5 were all compatible with the scenario that their founding haplotypes were present and directly involved in the domestication event that occurred 10–11 ky ago in the Near East. The exception was T4 whose younger age is suggestive of an origin within domestic cattle, probably while diffusing from the Near East towards Eastern Asia [22, 23].

Analyses of entire mitogenomes have also shown that haplogroups T1-T5 do not represent the totality of taurine mtDNAs. Three haplogroups, termed P, Q and R, all radiating prior to the T node in the taurine portion of the mtDNA phylogeny, have been detected in modern breeds [22–24], while two haplogroups, termed E and C, have been reported only in ancient specimens and are probably extinct. Haplogroup E was identified in a ∼6 ky old aurochs from Germany [14, 30] while haplogroup C was found in a specimen that might represent an early Holocene attempt to manage cattle in northern China [31].

The detection of haplogroup P, Q and R mtDNAs in modern individuals has fueled the debate on the origin of taurine cattle, and more generally on the role played by local aurochsen populations in its current genetic make-up. While there is very little doubt that the uncommon haplogroups P and R are derived from European wild aurochs cows either because of sporadic interbreeding events (naturally occurring and/or human-mediated) [22, 32] or possibly, in the case of haplogroup R, as consequence of a minor event of *B*. *primigenius* domestication in Italy [24], the origin of haplogroup Q is less clear.

Initially it was suggested that haplogroup Q might have derived from European aurochsen [22], while later a parallel history was instead hypothesized for haplogroups Q and T, with Q representing an additional lineage that was domesticated in the Near East and spread with trade and human migrations [23, 24]. Interestingly, a recent survey of prehistoric domestic cattle control-regions has identified haplogroup Q in Middle/Late Neolithic remains from Anatolia, and also at extremely high frequencies in skeletal remains from Bulgaria and Romania dated 7–4 ky ago [33]. Overall these findings highlight a growing complexity in the geographic distribution of Q, and lend support to the conclusion of Achilli and colleagues [23] who stated that the parallel history and source of haplogroups Q and T needed to be tested by acquiring coding-region data from a wide range of *B*. *taurus* populations, especially from the Near East. Unfortunately, despite the proven effectiveness of whole mitochondrial genome data in providing valuable information concerning the origin of taurine cattle, so far no population surveys have been carried out at the level of mitogenomes in local breeds from the Near East or surrounding areas.

In this study, to obtain more information concerning the mitogenome variation in a geographic area that is adjacent to the Near East, the postulated ancestral homeland of *Bos taurus* domestication, and thus possibly reconstruct some of the early events associated with the spread of domestic cattle out of the Near East, we analyzed a total of 31 mtDNA genomes (22 reported here for the first time; GenBank records KT184451- KT184472) from two autochthonous Egyptian cattle breeds (Menofi and Domiaty) (also known as Baladi and Damietta). Our analyses revealed an extremely high level of diversity in terms of haplotypes and haplogroup affiliation, which included haplogroup Q mtDNAs. Direct links and extensive genetic continuity with the domestic herds that first arrived from the Near East were identified, providing confirmation that haplogroups Q and T underwent domestication together in the Near East.

## Materials and Methods

### Ethics statement

All experimental procedures were reviewed and approved by the Animal Research Ethics Committee of the University of Pavia, Prot. 2/2010 (October 15^th^, 2010), in accordance with the European Union Directive 86/609.

### Samples

A set of 31 mtDNAs from the Nile Delta region were analyzed, 17 from the Menofi (Baladi) breed were sampled in the Menofia Governorate [Lat (30.59) Long (30.98)] and 14 from the Domiaty (Damietta) breed were sampled in the Damietta Governorate [Lat (31.36) Long (31.67)] (S1 Table). Both are members of the Humpless Shorthorn cattle type which is present in North Africa in two subgroups: one is the Egyptian, that includes the Baladi, Damietta, and Maryuti, from North West Egypt and the Nile Delta region, and the Saidi from Southern Egypt; the other is the Brown Atlas that is distributed along the wide coastal area encompassing Libya, Tunisia, Algeria and Morocco [34, 35]. DNAs were purified from peripheral blood according to standard methods. Nine of the 31 mitogenomes (5 Menofi and 4 Domiaty), all belonging to haplogroup T1, have been previously reported [27].

### Illumina sequencing of mitochondrial genomes

Twenty-two of the 31 Nile Delta mitogenomes were obtained in this study (S1 Table) by Next Generation Sequencing with an Illumina MiSeq^®^. A set of two overlapping PCR fragments (S2 Table) covering the entire mtDNA genome was used. The PCR protocol was as follows: 50 ng of each DNA sample were amplified in 50 μl reaction mixture containing 2.5 mM MgCl_2_, 0.4 mM of each dNTP (Takara), 1X LA PCR Buffer II (Mg^2+^ free, Takara), 2.5 U of LA Taq DNA Polymerase (Takara) and 0.6 μM of each primer. The PCR program included an initial denaturation step at 94°C for 1 minute followed by 30 cycles characterized by the following thermal profile: 98°C for 10”, 68°C for 15 minutes and a final extension step at 72°C for 10 minutes.

The two PCR products were purified with Wizard® SV Gel and PCR Clean-Up System (Promega) and quantified with a Quantus™ Fluorometer (Promega). A total amount of 1.5 ng of PCR product (0.75 ng for each PCR) were used for the set up of a sequencing library with the Nextera^®^ XT DNA sample preparation kit (Illumina). We followed the manufacturer's protocol, i.e. tagmentation of input DNA, amplification of tagmented DNA with the addition of indexes of the Nextera® XT Index Kit (24 Indices, 96 Samples), PCR clean-up with Agencourt^®^ AMPure^®^ XP (Beckman Coulter), library normalization, library pooling and MiSeq sample loading.

Sequencing reactions were carried out on a MiSeq (Illumina), by using the MiSeq Reagent Nano Kit, v2 (300 cycles). On-board software creates results in FASTQ format, which were then used to generate tab-delimited text files containing sequence alignment data (SAM files) or their binary version (BAM files) comparing them with the bovine reference sequence (BRS) [36] (GenBank ID V00654). The depth of the obtained reads was generally >100X. The Integrative Genomics Viewer (IGV) free software was used to align BAM files to the BRS and create a report of sequence variants (nucleotide substitutions and indels).

Three mitogenomes (#6, #30 and #31 in S1 Table) were also sequenced with the standard dideoxy sequencing [22] in order to assess the sequencing quality and reliability. In all cases the calling of variants was in total agreement with the outcome of the Illumina MiSeq^®^ sequencing.

### Coalescence and expansion times

Coalescence times were estimated using both maximum likelihood (ML) and the ρ statistic (average distance of the haplotypes of a clade from the respective root haplotype) [37] accompanied by a heuristic estimate of the standard error (σ) calculated from an estimate of the genealogy [38]. PamlX 1.3.1 [39] was used to obtain ML estimates, assuming the HKY85 mutation model (two parameters in the model of DNA evolution) with gamma-distributed rates (approximated by a discrete distribution with 32 categories) on the coding region (from np 364 to np 15791). These calculations were performed considering all the nucleotide substitutions (excluding heteroplasmies) in the coding region. Mutational distances were converted into years using the substitution rate for the bovine coding region of about one mutation every 3,172 years [22].

We also obtained Bayesian skyline plots (BSPs) [40] from BEAST 1.7.4 [41] for the 31 Egyptian mitogenomes with a relaxed molecular clock (lognormal distribution across branches and uncorrelated in between) and a HKY85-type model with γ-distributed rates to estimate effective population size through time. To approximate the mutation rate to the one used in previous analyses, a P sequence (GenBank ID DQ124389) was used as an outgroup [22], and an age for macro-haplogroup PQT of 75.5 ± 10.0 ky [24] was considered as a consistent internal calibration point. Specifically, we ran 100,000,000 iterations, with samples drawn every 10,000 Markov chain Monte Carlo (MCMC) steps, after a discarded burn-in of 10,000,000 steps, as in Soares et al. [42]. We considered haplogroup PQT and major subclades in our samples as monophyletic in the analyses. We visualized the BSPs obtained in plots with Tracer v1.5 and then converted them to Excel graphs by using a generation time of six years [43].

## Results and Discussion

### The phylogeny and diversity of Egyptian mitogenomes

**Fig 1:**
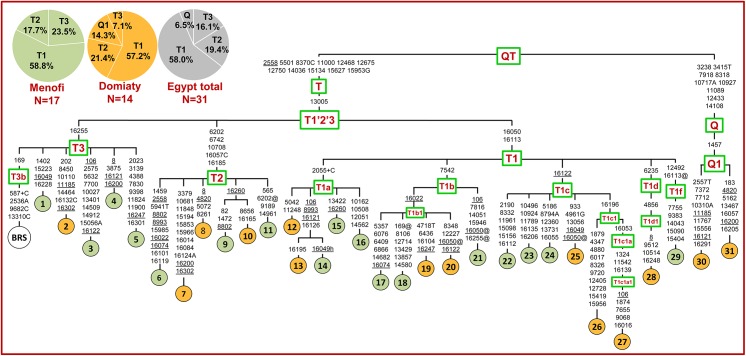
Tree of mitogenomes from Egyptian cattle. Sequences #1–19, #25, #30–31 have been determined in this study, while sequences #20–24 and #26–29 were previously reported [27]. GenBank accession numbers are reported in S1 Table. This tree was built as previously described [22–24, 27]. The hypervariable insertion of a G at np 364, the length variations in the C tract scored at np 221 and the A tract scored at np 1600 were not used for the phylogeny construction. The position of the Bovine Reference Sequence (BRS) [36] is indicated for reading off-sequence motifs. Branches display mutations with numbers according to the BRS; they are transitions unless a base is explicitly indicated for transversions (to A, G, C, or T) or a suffix for indels (+, d) and heteroplasmy (h). Recurrent mutations within the phylogeny are underlined and back mutations are marked with the suffix @. Note that the reconstruction of recurrent mutations in the control region is ambiguous in a number of cases. The pie charts summarize haplogroup frequencies in the Menofi (green) and Domiaty (orange) breeds.

The phylogenetic relationships of the 31 Egyptian mtDNAs are illustrated in Fig 1. As expected for African cattle [7], haplogroup T1 (58.0%) is the most frequent. However, in contrast to virtually all African breeds from other areas where haplogroup T1 is essentially fixed [11, 28, 44, 45], probably due to a number of sequential founder events that occurred as domesticated cattle spread from north to south across Africa [28], two other members of the macro-haplogroup T were also found in the Nile Delta breeds, both at rather high frequencies: T2 (19.4%) and T3 (16.1%). Whereas, haplogroup T5, which was previously reported at low frequencies both in Europe and Iraq [22], was not found in the Egyptian sample. In addition to the T mtDNAs, two mtDNAs (6.5%) belonging to the rare haplogroup Q1 were observed in the Domiaty breed.

The presence of non-T1 mtDNAs in Egypt has been already reported [11], however, the overall frequency of ∼42% detected here in each of the two Nile Delta breeds (Fig 1) is the highest reported so far. Moreover, the two (very divergent) complete Q mtDNA sequences found in the Domiaty breed are the first obtained from non-Italian breeds.

The high diversity in terms of haplogroups is not the only feature of the Egyptian cattle, our (randomly collected) samples are also characterized by an extremely high haplotype diversity (H = 1.0 ± 0.008) with each mitogenome harboring a different haplotype (Fig 1). Furthermore, almost all haplotypes depart directly from the respective haplogroup root, with a star-like topology and a rather similar average number of nucleotide differences within each haplogroup: T1 (M = 11.9), T2 (M = 11.7), T3 (M = 13.6) and Q1 (M = 16.0).

Currently these levels of diversity cannot be compared with those of breeds from other geographic areas. Indeed, only a rather limited number of cattle mtDNAs have been analyzed at the level of entire mitogenomes, and often the completely sequenced mtDNAs were not randomly chosen, but rather selected taking into account both haplogroup affiliation and extent of the control-region variation. This is because the main objective of most studies was to define the phylogeny and the ancestral origin of haplogroups rather than the origin of specific populations [22–24, 27]. There is only one random population recently surveyed at the level of mitogenomes, the Nguni breed (N = 35) from South Africa. In that population sample, however, haplogroup T1 turned out to be fixed and 94% of the individuals were found to cluster within a single sub-haplogroup (T1b) [27, 28].

### Relationships of the Egyptian mitogenomes with those from other geographic areas

**Fig 2:**
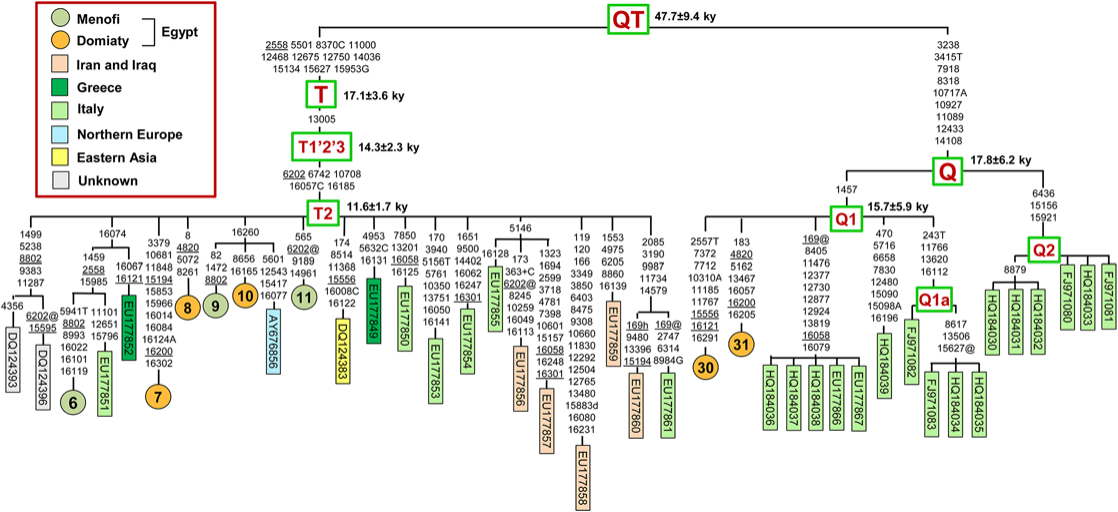
Worldwide phylogeny of taurine haplogroups T2 and Q. This most parsimonious tree encompasses the Egyptian mitogenomes belonging to haplogroups T2 (N = 6) and Q1 (N = 2) and all previously published worldwide mitogenomes from the same haplogroups (T2, N = 17 and Q, N = 16). Branches display mutations with numbers according to the BRS; they are transitions unless a base is explicitly indicated for transversions (to A, G, C, or T) or a suffix for indels (+, d) and heteroplasmy (h). Recurrent mutations within the phylogeny are underlined and back mutations are marked with the suffix @. Coalescence times are maximum likelihood (ML) estimates.

In order to further assess the phylogenetic relationships of the Egyptian cattle mitogenomes, in particular the possible links with those from other geographic areas, we compared their sequence variation with all available mitogenomes (from the same haplogroup) from public databases. The phylogenetic relationships of T2 and Q mitogenomes are illustrated in Fig 2, while those of the T3 mitogenomes are shown in S1 Fig This comparison is illustrated with detailed figures only for haplogroups Q, T2 and T3 because the phylogeny of T1 has been reassessed recently [27, 28].

The two Egyptian Q1 mtDNAs do not share non-founding mutations with Q1 mitogenomes from other geographic areas, while mutations are shared by three of the T2 mtDNAs (#6, #9 and #10) and one T3 mtDNA (#1). For three of these (T3 #1, T2 #9 and T2 #10) the mutational sharing is with two mitogenomes from Northern Europe (AY676856 from a Limousin and KC153972 from a White Park), and is restricted to a single control-region mutation that, in the case of the T3 #1, is recurrent (the transition at np 16049). Because of their location in the control-region, it is unclear whether these shared mutations mark ancient genetic links between mitogenomes that are now distant both in terms of breed and geography or whether they are the product of independent mutational events, with no real phylogenetic significance. A similar conclusion is true for three of the Egyptian T1 mitogenomes (#13, #14 and #15). They also share recurrent control-region mutations with previously published T1 mitogenomes (data not shown). The control-region transition at np 106 present in the T1a sequences #13 and #14 (Fig 1) has been reported in four T1a mitogenomes from Italian breeds (Chianina, Italian Podolian, Romagnola), while the T1a sequence #15 shares the control-region transition at np 16260 with a T1a mitogenome from an Italian Brown [27].

The phylogenetic link of the T2 mitogenome #6 is much more robust (Fig 2). In addition to six private mutations, it harbours four transitions (nps 1459, 2558, 15985 and 16074) previously reported in one mitogenome (EU177851) from a local cattle breed (Cabannina) of Liguria, a coastal region of northern Italy. This finding is compatible with historical evidence of ancient human migrations and sea trade networks in the Mediterranean basin [9, 46].

### Age estimates of taurine haplogroups and population expansion of Egyptian cattle

**Fig 3:**
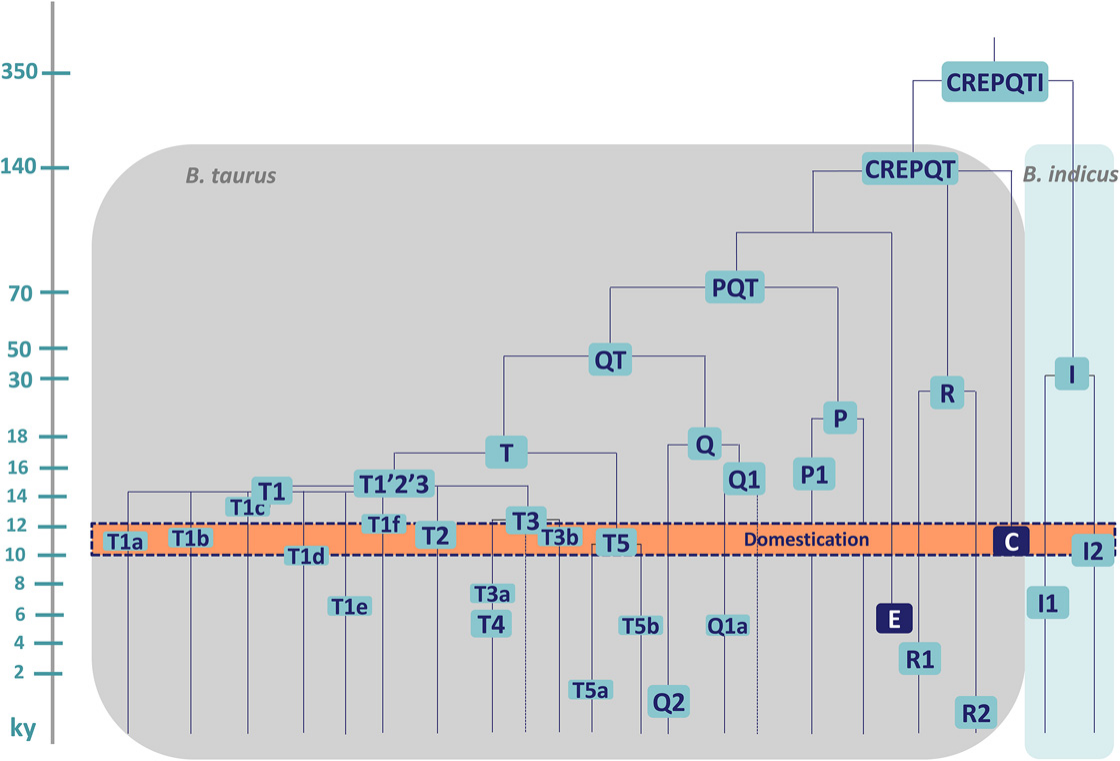
Schematic representation of the cattle mtDNA phylogeny. This tree highlights the founding haplotypes that most likely were involved in the domestication process. Approximate ages (ky) can be inferred from the scale. Some correspond to the ML ages in Table 1, those for haplogroups R and I are from [23, 24, 27], while those for the probably extinct haplotypes E and C correspond to the radiocarbon dates of the specimens in which they have been found [14, 31]. A dotted line is shown in T3 and Q1 to indicate that other not yet identified founder sub-haplogroups are likely for these two haplogroups.

Age estimates obtained with maximum likelihood (ML) and ρ statistics (Table 1) are rather similar for all haplogroups and they are in line with those reported in the past [22–24, 27]. Haplogroups T1, T2, T3, T5 and Q1 harbour ML coalescence times in the 11–15 ky range, thus they are in full agreement with the scenario that they were present in the Near Eastern aurochs population(s) that underwent domestication about 10 ky ago, especially when considering the possibility that the estimated ages of haplogroup nodes might be slightly inflated by purifying selection, as previously shown for humans at similar time depths [47]. The finding that for some of these haplogroups, specifically T1 and Q1, the coalescence ages predate the postulated domestication time by 4–5 ky, suggests that a certain degree of sequence diversity already existed within each haplogroup in the wild population(s) at the time of domestication, and more than one haplotype per haplogroup might have been involved in the domestication event(s). A full assessment of this scenario is premature for Q1, due to both the limited number of available mitogenomes and the bias towards mitogenomes from Italy, but it is compatible with the numerous haplotypes proposed as founders for modern haplogroup T1 mtDNAs [27]. A schematic overview of the taurine portion of the cattle mtDNA phylogeny highlighting all haplotypes that have been proposed as founders is depicted in Fig 3.

**Table 1:** Molecular divergence and age estimates (ML and ρ statistics) for taurine cattle haplogroups based on all currently available mitogenomes.

Haplogroup	N^a^	ML^b^	SE	T (ky)^c^	± ΔT (ky)	ρ	σ	T (ky) ^c^	± ΔT (ky)
PQT	273	24.04	3.78	76.24	11.99	15.5	3.08	49.17	9.77
>P^d^	3	6.51	1.79	20.65	5.68	7.33	1.70	23.25	5.39
>>P1^e^	2	4.97	1.56	15.76	4.94	6.00	2.24	19.03	7.11
>QT	270	15.04	2.95	47.71	9.35	15.59	3.12	49.45	9.90
>>Q	18	5.60	1.96	17.76	6.22	5.56	1.22	17.64	3.87
>>>Q1	12	4.94	1.87	15.66	5.92	6.08	1.34	19.29	4.25
>>>>Q1a	4	1.82	2.85	5.77	9.05	2.25	1.30	7.14	4.12
>>>Q2	6	0.29	6.20	0.93	19.67	0.50	0.50	1.59	1.59
>>T	252	5.38	1.13	17.08	3.57	5.67	1.05	17.99	3.33
>>>T1’2’3^f^	248	4.52	0.71	14.34	2.25	4.67	0.37	14.81	1.17
>>>>T1	107	4.46	0.97	14.14	3.08	4.91	0.70	15.57	2.22
>>>>>T1a	35	3.36	0.51	10.67	1.61	3.06	0.37	9.71	1.17
>>>>>T1b	45	3.53	1.05	11.21	3.33	5.91	1.28	18.75	4.06
>>>>>>T1b1	43	3.18	0.86	10.08	2.74	5.93	1.33	18.81	4.22
>>>>>T1c	17	4.46	1.23	14.14	3.92	4.00	0.58	12.69	1.84
>>>>>>T1c1	11	4.46	1.11	14.14	3.52	4.27	0.79	13.54	2.51
>>>>>>>T1c1a	5	4.46	1.59	14.14	5.04	3.60	1.30	11.42	4.12
>>>>>T1d	6	3.15	0.83	9.98	2.64	3.00	0.82	9.52	2.60
>>>>>>T1d1	3	2.05	0.76	6.51	2.40	2.00	0.82	6.34	2.60
>>>>>T1e	2	1.99	1.42	6.31	4.50	1.00	0.87	3.17	2.76
>>>>>T1f	2	3.92	0.97	12.43	3.08	5.50	1.66	17.45	5.27
>>>>T2	23	3.66	0.52	11.60	1.66	4.30	0.49	13.64	1.55
>>>>T3	117	3.93	0.49	12.48	1.57	3.95	0.26	12.53	0.82
>>>>>T3b	25	3.61	0.65	11.45	2.06	3.68	0.47	11.67	1.49
>>>>>T3a	10	2.47	1.31	7.83	4.16	2.70	0.92	8.56	2.92
>>>>>>T4	7	1.79	2.59	5.68	8.22	2.29	0.61	7.26	1.93
>>>>T5	4	3.38	1.06	10.72	3.38	3.50	1.15	11.10	3.65
>>>>>T5a^g^	2	0.42	0.42	1.32	1.32	0.50	0.50	1.59	1.59
>>>>>T5b^g^	2	1.82	0.96	5.77	3.03	1.50	0.87	4.76	2.76

^a^ Number of mitogenomes. For haplogroups T2, Q and T3, the mitogenomes correspond to those reported in Fig 2 and S1 Fig. Haplogroup T1 includes mitogenomes from this study (S1 Table and Fig 1) and from the literature [27, 28], while T5 mitogenomes are those from [22].

^b^ Maximum Likelihood molecular divergence.

^c^ Age estimates (ky) using the molecular clock proposed by Achilli et al. [22].

^d^ Haplogroup P includes three published mitogenomes (NC013996, JQ437479, DQ124389).

^e^ Subclade P1 has been defined here and includes mitogenomes NC013996 and DQ124389.

^f^ Haplogroup T1’2’3 includes the EU177840 mtDNA sequence [22], in addition to the T1, T2 and T3 mitogenomes.

^g^ Haplogroups T5a and T5b have been defined here.

**Fig 4:**
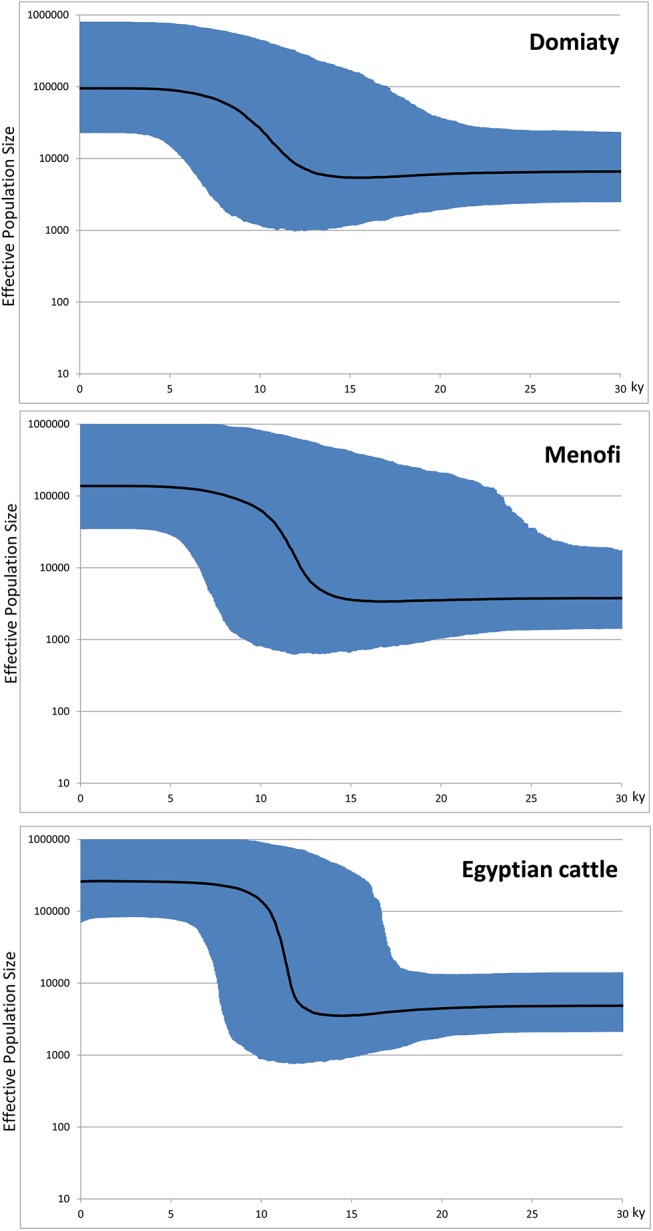
Bayesian Skyline Plots showing the size trend of the Egyptian cattle. The top BSP refers to the Domiaty sample (N = 14), the central one to the Menofi sample (N = 17), while the lower BSP was obtained by considering both samples. The Y axis indicates the effective number of females. The thick solid line is the median estimate and the blue shading shows the 95% highest posterior density limits. The time axis is limited to 30 ky, beyond that time the curve remains linear. A generation time of six years was employed [43].

To assess population expansions that might have involved the Nile Delta cattle, Bayesian Skyline Plots (BSPs) were obtained (Fig 4). The BSPs, obtained both on single breeds and on the whole Egyptian sample, point to a main episode of population growth beginning at ~12.5 ky ago that, taking into account the confidence intervals, probably reflects the initial population expansion that followed the domestication of wild aurochs (*Bos primigenius*) in the Near East, although a Late/postglacial expansion of wild cattle, prior to domestication, can not be ruled out.

### The origin of *Bos taurus* haplogroup Q

The debate on the origin of domesticated cattle, and more in general the role played by local aurochsen populations in its current genetic make-up, has received new fuel with the finding of haplogroup P, Q and R in modern individuals. While an ancestry from European wild aurochs is generally accepted for haplogroups P and R, the source of haplogroup Q is more controversial. With an estimated age of about 48 ky for the QT node (Fig 2), haplogroup Q is the closest to super-haplogroup T and the only non-T haplogroup that we found in the Egyptian cattle. Taking into account the limited size of the sample collection (31 subjects), the finding of two Q1 mtDNAs in the Domiaty breed, with very different haplotypes and each rooting directly from the Q1 node (Fig 1), is important for its implications for the debate concerning the ancestral source of haplogroup Q.

This haplogroup was first discovered in a local breed (Cabannina, two mtDNAs with the same haplotype) from Liguria (Italy) [22] and it was initially suggested that, similarly to the rare haplogroup P mtDNAs found in modern cattle, it might have derived from European aurochsen, possibly from a *Bos primigenius* population that might have ranged only south of the Alps [22]. Fourteen additional Q mtDNAs were identified and completely sequenced (Fig 2) in the following two years, but all of these were from Italian breeds (Cabannina, Chianina, Grey Alpine, Italian Red Pied, and Romagnola) [23, 24].

The same studies made attempts to obtain information concerning the presence of Q mtDNAs in other geographic areas and breeds by surveying published control-region sequences from worldwide samples despite two major limitations: (i) there is only one diagnostic mutation in the mtDNA control region, the C to G transversion at np 15953, distinguishing Q and T mtDNAs; (ii) the np 15953 is not included in many of the published control-region datasets [23]. The outcome of these surveys was that haplogroup Q was probably present in modern breeds from several European countries, Turkey and China as well as in cattle skeletal remains from Neolithic archeological sites in Germany, France, and Eastern Thrace (Turkey). In view of these findings, the initially envisioned origin of Q from European aurochs was reconsidered, and a parallel history was hypothesized for haplogroups Q and T, with Q representing an additional lineage that was domesticated in the Near East and later spread with trade and human migrations [24]. Interestingly, a very recent and extensive survey of prehistoric domestic cattle control-regions, mainly from Southeastern Europe and Western Anatolia, has revealed the presence of haplogroup Q in Middle/Late Neolithic bones from Anatolia, but also with surprisingly high frequencies in Bulgaria and Romania 7,500–7,000 years ago and 4,700–4,200 years ago (50% and 29%, respectively) [33].

In the present study, we detected and completely sequenced two Q mtDNAs from a geographic area that is not Italy, but the Nile Delta region, which is in close geographic and cultural proximity to the Near East, and was one of the first neighboring areas to adopt the Neolithic package, including cattle rearing. This finding has relevant implications for the debate concerning the ancestral source of haplogroup Q for a number of reasons.

First, the inclusion of the two Egyptian Q1 mitogenomes in the phylogeny allows for a more precise estimate of the Q and Q1 coalescence times (Fig 2). The current ML estimate of 15.7 ± 5.9 ky for Q1 indicates a rather recent (late glacial) origin, which makes it unlikely that this haplogroup had the time to spread in aurochsen populations living far away from each other. In particular, it is extremely unlikely that Q1 mtDNAs were present in the *Bos primigenius* populations of both Europe and the Near East prior to the Neolithic transition. Second, the detection of two Q mtDNAs in a local breed (Domiaty) from Egypt weakens the scenario that the ancestral source of haplogroup Q might have been European aurochs, unless gene flow in historical times from European domestic cattle is envisioned. However, if this gene flow had occurred, it should have mainly involved haplogroup T3, which represents the vast majority of European mtDNAs [44], a scenario that does not appear compatible with the haplogroup composition of the Domiaty breed where only 7.1% is represented by T3 mtDNAs. Third, despite the two Egyptian Q1 mtDNAs are from the same breed, they harbor different and extremely divergent haplotypes (#30 and #31), which radiate directly from the Q1 node (Fig 2) and do not share any of their private mutations with any of the Q1 mitogenomes reported so far (all from Europe). This again does not support gene flow from Europe, but rather suggests that the two Egyptian Q1 mtDNAs are direct local derivatives from Q1 founder mtDNAs brought to Egypt by the first domestic herds. In other words, similar to T1, T2 and T3, Q1 was among the haplogroups involved in domestication in the Near East, from where it spread along with the others.

An *in situ* differentiation from the founder domestic herds which first arrived in Egypt from the Near East appears to be very likely not only for the Q1 mtDNAs but also for the majority of Domiaty and Menofi mitogenomes. Indeed, many of the T1, T2 and T3 mitogenomes depart from nodal haplotypes which most likely were directly involved in the domestication process (Fig 3). Moreover, they do not share private mutations with other mitogenomes of the same haplogroup from other geographic areas, suggesting that there was very limited gene flow towards and from the two Egyptian cattle breeds, at least on the maternal line, after the initial founder event. One exception to this general trend is represented by a T2 mitogenome (#6) that shares four transitions with a mitogenome from a local cattle breed (Cabannina) from Italy, a finding that indicates direct or indirect gene flow between the two geographic areas, probably as a result of trades or human migrations.

## Conclusions

Phylogenetic analyses of 31 Egyptian mitogenomes from two Nile Delta taurine breeds confirmed the prevalence of haplogroup T1, but also showed rather high frequencies for haplogroups T2, T3 and Q1, and an extreme haplotype diversity. Bayesian analyses revealed a main episode of population growth beginning at ~12.5 ky ago that is compatible with the earliest archeozoological evidence of bone size reduction in cattle derived from Dja'de el Mughara in the Middle Euphrates Valley [1], possibly marking the transition from wild *Bos primigenius* to domestic *Bos taurus* [3]. Thus, the population growth that we observe by analyzing Egyptian mitogenomes probably reflects the initial population expansion that followed the domestication of *Bos primigenius* in the Near East. Finally, comparisons of the Nile Delta mitogenomes with those available from other geographic areas show that most Egyptian mtDNAs are probably direct local derivatives of the founder domestic herds, which first arrived from the Near East and that, after the initial founder event, there was very limited gene flow, on the maternal lineages, towards and from other geographic areas, at least those areas from which complete mitogenomes are currently available. Most importantly, haplogroup Q1 mtDNAs were among these early founders, thus providing confirmation that macro-haplogroups Q and T underwent domestication together in the Near East.

## Supporting Information

S1 Fig Detailed worldwide phylogeny of cattle haplogroup T3. This most parsimonious tree encompasses the Egyptian mitogenomes belonging to haplogroup T3 (N = 5) and 112 previously published worldwide mitogenomes from the same haplogroup, including the BRS [36]. Branches display mutations with numbers according to the BRS; they are transitions unless a base is explicitly indicated for transversions (to A, G, C, or T) or a suffix for indels (+, d) and heteroplasmy (h). Recurrent mutations within the phylogeny are underlined and back mutations are marked with the suffix @. The reported T3 coalescence time is a maximum likelihood (ML) estimate.
(TIF)

S1 Table Origin and sub-haplogroup affiliation of mitogenomes from Egyptian cattle breeds considered in this study. (DOCX)

S2 Table Amplicons and oligonucleotides used for sequencing the whole bovine mitochondrial genome with the Illumina MiSeq®. (DOCX)
