# Supplementary figures and images for: Mitogenomes from Egyptian Cattle Breeds: New Clues on the Origin of Haplogroup Q and the Early Spread of Bos taurus from the Near East
Source: PLoS One. 2015 Oct 29;10(10):e0141170. doi: 10.1371/journal.pone.0141170 (PMC4626031; doi:10.1371/journal.pone.0141170)

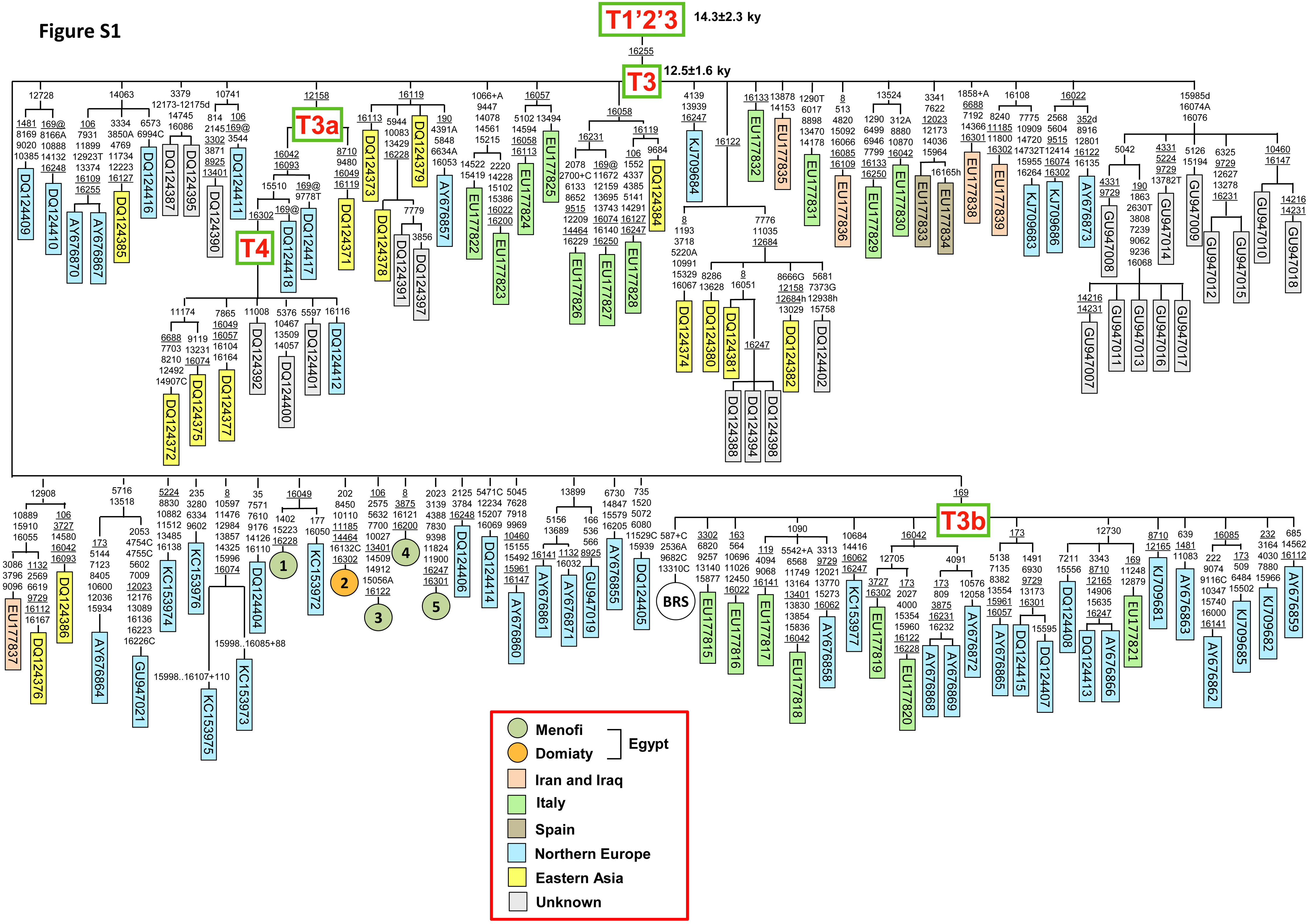

Supplement: S1 Fig — This most parsimonious tree encompasses the Egyptian mitogenomes belonging to haplogroup T3 (N = 5) and 112 previously published worldwide mitogenomes from the same haplogroup, including the BRS [36]. Branches display mutations with numbers according to the BRS; they are transitions unless a base is explicitly indicated for transversions (to A, G, C, or T) or a suffix for indels (+, d) and heteroplasmy (h). Recurrent mutations within the phylogeny are underlined and back mutations are marked with the suffix @. The reported T3 coalescence time is a maximum likelihood (ML) estimate. (TIF) [file pone.0141170.s001.tif]
