# Supplementary material for: Mitogenomes from Egyptian Cattle Breeds: New Clues on the Origin of Haplogroup Q and the Early Spread of Bos taurus from the Near East
Source: PLoS One. 2015 Oct 29;10(10):e0141170. doi: 10.1371/journal.pone.0141170 (PMC4626031; doi:10.1371/journal.pone.0141170)
Supplement: S1 Table — (DOCX) [file pone.0141170.s002.docx]

**S1 Table. Origin and sub-haplogroup affiliation of mitogenomes from Egyptian cattle breeds considered in this study.**

| **Sequence ID#** ^a^ | **Original Name** | **Breed** | **Haplogroup** | **GenBank ID** | **Reference** |
| --- | --- | --- | --- | --- | --- |
| 1 | EG35 | Menofi | T3 | KT184451 | This study |
| 2 | EG14 | Domiaty | T3 | KT184452 | This study |
| 3 | EG24 | Menofi | T3 | KT184453 | This study |
| 4 | EG39 | Menofi | T3 | KT184454 | This study |
| 5 | EG40 | Menofi | T3 | KT184455 | This study |
| 6^b^ | EG32 | Menofi | T2 | KT184456 | This study |
| 7 | EG6 | Domiaty | T2 | KT184457 | This study |
| 8 | EG7 | Domiaty | T2 | KT184458 | This study |
| 9 | EG38 | Menofi | T2 | KT184459 | This study |
| 10 | EG3 | Domiaty | T2 | KT184460 | This study |
| 11 | EG41 | Menofi | T2 | KT184461 | This study |
| 12 ^c^ | EG1 | Domiaty | T1a | KT184462 | This study |
| 13 ^c^ | EG2 | Domiaty | T1a | KT184463 | This study |
| 14 ^c^ | EG30 | Menofi | T1a | KT184464 | This study |
| 15 ^c^ | EG31 | Menofi | T1a | KT184465 | This study |
| 16 ^c^ | EG33 | Menofi | T1a | KT184466 | This study |
| 17 ^c^ | EG37 | Menofi | T1a | KT184467 | This study |
| 18 ^c^ | EG34 | Menofi | T1a | KT184468 | This study |
| 19 ^c^ | EG9 | Domiaty | T1a | KT184469 | This study |
| 20 ^c^ | EG19 | Domiaty | T1b1 | JN817324 | [27] |
| 21 ^c^ | EG27 | Menofi | T1b | JN817327 | [27] |
| 22 ^c^ | EG23 | Menofi | T1c | JN817326 | [27] |
| 23^c^ | EG22 | Menofi | T1c | JN817325 | [27] |
| 24 ^c^ | EG28 | Menofi | T1c | JN817328 | [27] |
| 25 ^c^ | EG5 | Domiaty | T1c | KT184470 | This study |
| 26 ^c^ | EG18 | Domiaty | T1c1 | JN817323 | [27] |
| 27 ^c^ | EG12 | Domiaty | T1c1a1 | JN817322 | [27] |
| 28 ^c^ | EG11 | Domiaty | T1d1 | JN817321 | [27] |
| 29 ^c^ | EG36 | Menofi | T1f | JN817329 | [27] |
| 30^b^ | EG4 | Domiaty | Q1 | KT184471 | This study |
| 31^b^ | EG10 | Domiaty | Q1 | KT184472 | This study |

^a^ ID numbers are those reported in the phylogeny of Fig. 1.

^b^ Completely sequenced also with the Sanger approach.

^c^ All T1 mitogenomes were double-checked for the presence of the insertion (G or A) at np 16201 recently reported by Horsburgh et al. [28]. In agreement with Bonfiglio et al. [27], none was found to harbor it.
