# Supplementary material for: Mitogenomes from Egyptian Cattle Breeds: New Clues on the Origin of Haplogroup Q and the Early Spread of Bos taurus from the Near East
Source: PLoS One. 2015 Oct 29;10(10):e0141170. doi: 10.1371/journal.pone.0141170 (PMC4626031; doi:10.1371/journal.pone.0141170)
Supplement: S2 Table — (DOCX) [file pone.0141170.s003.docx]

**S2 Table. Amplicons and oligonucleotides used for sequencing the whole mitochondrial genome with the Illumina MiSeq®.**

| **Fragment #** | **PCR product length (bp)** | **Oligonucleotides 5’-3’^a^** | |
| --- | --- | --- | --- |
| 1 | 8661 | 1801 For | TGGTGATAGCTGGTTGTCCA |
|  |  | 10461 Rev | GACCTAGGGCTGCTTCACAG |
| 2 | 8655 | 9920 For | GCCACCCTACTCGTCATCAT |
|  |  | 2236 Rev | TGCACTCCTGTGTTGGGTTA |

^a^ The oligonucleotide codes refer to the nucleotide position of the initial (5’) base; For, forward; Rev, reverse.
